# Development of a core competence model for improving medical college students’ ability in respond to public health emergencies

**DOI:** 10.3389/fpubh.2025.1467832

**Published:** 2025-03-20

**Authors:** Fengqiong Liu, Shuqian Huang, Fa Chen, Suhua Yang, Lixin Wu, Yulan Lin, Baochang He, Zhijian Hu

**Affiliations:** ^1^Fujian Provincial Key Laboratory of Environment Factors and Cancer, Department of Epidemiology and Health Statistics, School of Public Health, Fuzhou, Fujian, China; ^2^School of Public Health, Fujian Medical University, Fuzhou, Fujian, China

**Keywords:** medical education, competency, framework, public health emergencies, undergraduate

## Abstract

**Background:**

Core competences has been developed for public health professionals worldwide. However, there is no core competences framework as to how to evaluate public health professionals and undergraduate students’ ability to respond to public health emergencies.

**Objectives:**

To develop a framework of core competences in public health emergencies for education of medical college student who majored in public health. To assess the knowledge and skill level of undergraduate students with public health background in respond to health emergency event and get to known the training needs.

**Methods:**

The Delphi method was applied to develop an agreed list of competences, which was followed by the construction of a competences framework in public health emergencies. A questionnaire consists of items that was derived from the list of competency statements developed by Delphi scoring to quantitatively report the knowledge and practical skill level and training needs of medical college students of public health background in public health emergencies.

**Results:**

An agreed set of core competences containing 43 statements was derived from the first and second Delphi scoring steps which can be grouped into four domains: intellectual competences, practical competences, behavioral competences, personnel and motivation. A total of 441 undergraduate students with public health background participated in the survey. The average performance of intellectual and practical competences is 3 in a 5-point scale, which equals 60 converted to 100 points. A better performance was observed in indicators of behavioral competences and personnel and motivation with an average score of 4 for most of the items, which equals 80 converted to 100 points. Admission year and sex are significantly related to competency performance of all domains with *β* value of −0.141 (*p* = 0.003) and − 0.237 (*p* < 0.001) for the overall performance.

**Conclusion:**

A framework of 43 core competences was developed, which covered both technical and general competencies in public health emergencies and represent the current competence demands of public health work force to be qualified for their job roles in public health emergencies for the local government in Fujian province. The concordance rate regarding to score of importance of the core competences are all >80% in both the first and second round Delphi survey, suggesting considerable reliability of the framework.

## Introduction

Great attention has been brought to public health emergencies globally since the COVID-19 pandemic (1). Public health emergencies refer to outbreaks of major infectious diseases, clusters of cause-unknown diseases, major food or occupational poisoning and other sudden incidents that could severely endanger public health. An Effective responses can greatly reduce the damage caused by public health events (2). Public health events often occur suddenly and unpredictably. Moreover, events may emerge in different forms in the future (3). Therefore, it is of great urgency to improve the ability in health emergency prevention and control. Medical workers provide healthcare practice and conduct case investigation and control in public health emergencies, which makes them of crucial importance in disease prevention and control (4, 5).

The COVID-19 pandemic revealed the inadequacy of a ready, willing and able workforce for health emergency work, which was attributed to the defects and deficiencies in medical training (6). Widespread educational reform has been implemented in most schools in China. Knowledge and skills of public health has been intensified in medical students’ curricula training program (7). Competence of adaption to outbreak of unpredictable public health events has been emphasized in educational programs of healthcare professionals, especially in medical specialties of public health (8). And existing curricula are trying to fill gaps in competencies of existing personnel and the urgent need to prepare an adequate surge capacity for emergency work.

Great effort has been made to develop core competences for public health professionals worldwide (9–13). However, the existing core competences frameworks are not specifically designed for the emergency education of medical students at school. For example, the “ASPHER’s European List of Core Competences for the Public Health Professional,” “Public Health Skills and Knowledge Framework 2019”released by United Kingdom, “Core Competencies For Public Health: a Regional Framework for the Americas 2013”released by United States, are all frameworks proposed for the general public health personnel. Some of the content of these framework refer to emergency response, but do not systematically address public health emergencies issues. The “WHO Health Emergencies Program Learning Strategy” has been developed to address health emergencies training. However, the program specifically applies to health workforce instead of medical students on campus.

There has been very limited data analyzing the current situation of in public health emergencies education and systematically discussing this issue. And there is no core competences framework as how to measure and evaluate public health professionals, as well as undergraduate’ performance in public health emergencies in China. The aim of this study is to develop an agreed set of core competences of public health emergencies for medical college students who majored in public health and will be health professionals in public health. We also sought to track the learning and training effect, and to understand the achievement of competencies of undergraduate students in emergencies, as well as the student’s training demands. The identified core competences provide guidance and reference for learning and training activities of medical students in public health. Our findings can serve as the overarching framework to achieving targeted and practical education for future medical undergraduate students.

## Methods

### Development of an initial list of core competences

We employed the multi-step interactive Delphi method that collect opinions of experts through questionnaires face-to-face interview.

Firstly, we drafted an interview outline and conducted a narrative review with five public health experts, to review the core competency frameworks globally and generate an initial list of competencies. The selected interviewees are the personnel in charge of public health emergency from the five largest Disease Control Center, who are experienced health workers and familiar with the public health emergencies management system in Fujian province.

The available core competency frameworks examined include “Core Competencies For Public Health in Canada 2008” (14), “Public Health Skills and Knowledge Framework 2019” released by United Kingdom (15), “Core Competencies For Public Health: a Regional Framework for the Americas 2013” released by United States (16),“WHO Health Emergencies Program Learning Strategy” (17). And the competences listed for China MPH curriculum were also assessed. In reviewing the similarities and differences across the frameworks, we merged domains and competencies that were similar and selected dissimilar ones that we deemed valuable in the local context to be considered by the Delphi participants. The Delphi respondents underscored the importance of a variety of competencies, and to identify context-specific core competencies to consider.

In addition to the selection of competency statements from existing competency frameworks, we also asked respondents about their understanding of the current emergencies management system competencies of existing personnel and targeted competency demands of local government. Critic public health services that are expected to provide, as well as public health responsibilities that health workforce are expected to fulfill were reviewed. We interviewed about the expert’s understanding of competencies, and the essential competencies required in the delivery of public health services in local government. Moreover, program through which public health workforce acquire targeted competencies was discussed, and understanding gaps between the current workforce development efforts and the current systems. Findings from these questions were incorporated in the results of existing competency frameworks by either expanding on or retaining competency items.

We then reworked the competency statements to integrate the findings of all the interviews after the narrative review and key informant interviews and to develop an initial list of 46 competency across four domains.

### Delphi scoring and analysis

To further justify the selection of competency statements and make the competency statements framework more generalized, we arrive at a set of public health competencies through a consensus-building process that included a wide range of professionals from the local system, as this would likely increase the prospects of the results being used in workforce development. Two round of Delphi surveys were subsequently undertaken to delineate and refine the list of competences. Interviewees have extended experience in the training program of public health emergencies and familiar with public health work force were invited.

A diverse group of 21 professionals with expertise in medical education and public health system in Fujian province of China participated in the meeting. Among the 21 participants, 14 experts are the responsible person of public health emergency from 14 Disease Control Center (CDC), which covered most of the CDC agency in Fujian province. Three experts are employees from three Community Health Service Center, which are the largest service center in Fuzhou City, capital of Fujian province. Four experts are employees for Fujian Medical University, which is the largest medical university in Fujian province. The demographic characteristics of the professionals was presented in Table 1. The expert panel cannot cover all the emergency related agency and personnel in Fujian province, so may introduce potential selection biases and cannot reflect yield a full picture of current emergency training needs for local government. However, CDC and Community Health Service Center are the most important unit in emergency response procedure, and the most experienced experts we selected from this agencies can be of reasonable representativeness.

**Table 1:** Demographic characteristics of the Delphi participants.

Demographic characteristics/Variable	Number (%)
Gender	
Male	14 (67)
Female	7 (33)
Place of work	
Community health service center	3 (14)
Centers for disease control and prevention	14 (67)
Medical university	4 (19)
Position	
Department of emergency management	8 (38)
Department of information processing	1 (5)
Department of infectious disease control and prevention	6 (28)
Department of inspection and quality control	1 (5)
Department of health management	1 (5)
School of public health	4 (20)
Specialty	
Microbiology	1 (5)
Public health administration	2 (10)
Clinical medicine	3 (14)
Lab medicine	3 (14)
Epidemiology and health statistics	3 (14)
Occupational health and environmental hygiene	1 (5)
Preventive medicine	8 (38)
Educational background	
Bachelor’s degree	9 (43)
Master’s degree	4 (19)
Doctor’s degree	5 (24)
Others	3 (14)

The 21 experts reviewed the list of competency and individually ranked each of the 46 items according to importance on a five-point Likert scale. A scale of one represent “not at all important” while a scale of five represent “absolutely essential.” The results from the investigation were summarized and competency items were ranked from high to low importance by medians. Results from the first round of Delphi scoring were discussed among the 21 experts, and the main points of modification was reported back. To complete the second round of Delphi, the optimized list of competency was returned to the 21 interviewees who scored these statements again.

### Investigation of the competences of public health emergencies in medical students in China

A questionnaire consisting of items that was derived from the list of competency statements developed by Delphi scoring to assess the knowledge and skill level of undergraduate students in respond to health emergency event and get to known the training needs.

The questionnaire contains two parts. In part one, item 1–5 shows basic characteristics of study participants. In part two, item 6–29 measures four scales of competency statements, which consisting of intellectual competences, practical competences, behavioral competences, personnel and motivation. The four scales of competency statements were measured by items 6–7, 8–10, 11–24, 25–29, respectively.

Scores were calculated separately for the four competency section. Higher score indicates greater competency potential in public health emergencies. Questions was evaluated individually on a 5-point scale ranging from 1 to 5 where 1–5 indicated very insufficient, insufficient, sufficient, good and very good performance in competency. The total score is a sum of the scores of all four domains which ranges from 24 to 120.

A total of 441 medical college students with a public health background from the Fujian medical University from Southeast China, completed the investigation. All participants were undergraduate with speciality in preventive medicine, public health administration, health inspection and quarantine. Participants were selected by random sampling according to their subject at university and admission year. The proportion of sampling were 20, 40, and 60% for the admission year of 2018, 2019, 2020, respectively. A total of 460 questionnaire was anonymously administered to the students who voluntarily completed the survey. The response rate was >90% and 441 questionnaires were available. The study sample cannot represent all the students, so may introduce selection bias. However random sampling was applied to help reduce selection bias and improve the representiveness of the sample.

The survey was anonymously administered to the students who completed the survey voluntarily and consent was obtained. The proposal for this study was approved by the Institutional Review Board (IRB) of Fujian Medical University (Project no: J22005).

### Data analysis

All data were analyzed using the software R (version 3.1.1). The stability between two rounds of Delphi scoring was assessed using the Wilcoxon signed-rank test. A competency statement was considered reliable if no statistically significant change was observed between the two round of Delphi score (*p* ≥ 0.05). The concordance rate was calculated to assess the consensus of the survey A competency statement was considered to be of acceptable consensus if the rating is 4 (very important) or 5 points (absolutely essential) by over 80% of the interviewees in the Delphi scoring.

Descriptive statistics were calculated to show the characteristics of the variables. The Mann–Whitney *U* test was used to test the distribution of scores across different factor groups. Linear regression analysis was used to explore the potential determinants of competency scores. The level of statistical significance was set at *α* = 0.05.

## Results

### Findings from the Delphi survey

Intellectual competences, practical competences, behavioral competences, personnel and motivation were highlighted as important competency areas. The initial list of competences contains 46 competency statements from four domains (Table 2). The intellectual competence domain include courses and disciplines, standards and regulations, which consisting of 10 indicators. This domain demonstrates knowledge about epidemiology, biostatistics, microbiology, infectious disease, food hygiene and environmental health, advances in public health, as well as knowledge about laws, regulations, guidelines and standards related to public health emergencies. The practical competences include field investigation, information management, epidemic situation judgment, which consisting of 15 indicators. This domain underscored the importance of a variety of competences in outbreak investigation, including being familiar with the steps and skills of an outbreak investigation, understanding of risk analysis frameworks and the determinants of risk assessment, management and communication. Behavioral competences include professionalism, comprehensive quality, emotion, attitude and value, which consisted of 15 indicators. Emergencies personnel must be able to effectively perform in rapidly changing, highly stressful and often insecure emergencies contexts, and require a great deal of flexibility, adaptability, resilience, innovation, being situationally aware, having a good contextual understanding, communicating and collaborates, with the highest professional and ethical behavior and commitment. The domain of personnel and motivation includes seven indicators, which put emphasize on innovation, actively learns, responsibilities, professional identity.

**Table 2:** Health emergencies competency framework for student cultivation in public health and results from Delphi scoring.

Domain	List of competency	Indicators	Importance^1^		Importance^2^		*P*
			Median	Consensus(%)	Median	Consensus(%)	
1. Intellectual competences	1.1. Courses and disciplines	Epidemiology	5	18 (100)	5	15 (100)	>0.5
		Biostatistics	5	19 (95.00)	5	15 (100)	0.655
		Microbiology	5	21 (100)	5	14 (93.33)	0.317
		Infectious disease	5	21 (100)	5	15 (100)	>0.5
		Food hygiene and environmental health	4.5	18 (90.00)	5	15 (100)	0.102
		Other medical related knowledge	3.5	12 (60.00)	–	–	–
		Advances in public health	4	16 (80.00)	4.5	15 (100)	0.046
	1.2. Standards and regulations	Laws	4.5	20 (100)	5	13 (92.86)	0.317
		Regulations	4	21 (100)	4.5	15 (100)	0.564
		Guidelines and Standards	5	19 (100)	5	15 (100)	>0.5
2. Practical competences	2.1. Field investigation	Survey preparation	5	19 (100)	5	15 (100)	>0.5
		Self-protection	5	20 (100)	5	15 (100)	0.317
		Case investigation	5	19 (100)	5	15 (100)	0.083
		On-site hygiene survey and risk evaluation	5	20 (95.24)	5	15 (100)	0.317
		Sample collection and transportation/delivery	5	18 (94.74)	5	14 (93.33)	0.564
		Interpretation of test results and test guidance	5	20 (95.24)	5	14 (93.33)	0.317
		Targeted and feasible measures for disease control	5	19 (90.48)	5	15 (100)	0.655
	2.2. Information management	Familiar with varied information management system	5	17 (85.00)	5	15 (100)	0.725
		Data analysis and management	5	18 (90.00)	4.5	14 (93.33)	>0.5
		Use of statistical software	4.5	17 (85.00)	5	14 (93.33)	0.414
		Report writing	5	19 (95.00)	5	15 (100)	0.317
	2.3. Timely carry out the epidemic situation judged	Identification of disease causality and chain of transmission in the region	5	20 (95.24)	5	15 (100)	0.317
		Origin-tracing of the outbreak	5	18 (95.71)	5	13 (86.67)	0.059
		Prediction of Epidemic Spread of the outbreak	5	19 (95.00)	5	15 (100)	0.317
		Perform a risk assessment and continuously review	5	20 (100)	4.5	15 (100)	0.564
3. Behavioral competences	3.1. Professionalism	Flexibility, agility, and adaptability	5	16 (88.89)	5	15 (100)	0.102
		Situational awareness in diverse cultural environments	4	13 (72.22)	–	–	–
		Systems and critic thinking	5	19 (95.00)	5	14 (93.33)	>0.5
		Risk identification	5	17 (85.00)	5	15 (100)	0.102
	3.2. Comprehensive quality	Physical fitness	5	16 (84.21)	4.5	14 (93.33)	0.655
		Resilience, effectively manage stress	5	16 (88.89)	5	15 (100)	0.564
		Communications	5	18 (94.74)	5	15 (100)	0.655
		Execution	5	18 (94.74)	5	15 (100)	0.083
		Situationally aware and contextual understanding	5	16 (84.21)	5	14 (93.33)	0.655
		Teamwork and Partnership	5	18 (94.74)	5	15 (100)	>0.5
		Leadership	–	–	4.5	14 (93.33)	–
		Lecture and presentation	4	17 (85.00)	4.5	13 (86.67)	0.18
	3.3. Emotion, attitude, and value	Big picture thinking	5	17 (89.47)	5	15 (100)	>0.5
		Dedication for working	5	17 (89.47)	5	14 (93.33)	0.564
		Sense of discipline and boundaries	5	17 (89.47)	5	14 (93.33)	0.564
4. Personnel and motivation	4.1. Personnel	Proactive in work	5	15 (78.95)	–	–	–
		Carefulness in work	5	17 (89.47)	5	14 (93.33)	0.564
		Innovation	4	16 (84.21)	5	15 (100)	>0.5
		Actively learns	5	17 (94.44)	5	15 (100)	>0.5
	4.2. Motivation	Responsibilities	5	15 (83.33)	5	13 (86.67)	>0.5
		Professional identity	5	15 (83.33)	5	13 (86.67)	0.317
		Achievement motivation	5	13 (79.47)	–	–	–

1 Represent results from the first round of Delphi scoring.

2 Represent results from the second round of Delphi scoring.

Several changes were proposed after the first around of Delphi scoring and the amendments was shown in Table 2. In the behavioral competences domain, the indicator of leadership was added. Four indicators were removed from the domain of intellectual, behavioral competences and personnel and motivation because the consensus (%) is <80% in the first round of Delphi scoring.

A total of 42 statements was considered as reliable according to the two steps of Delphi scoring as shown in Table 2. The distribution score of one statement was found different between two round of Delphi scoring. However, the distribution difference is of marginal statistic significance with *p* = 0.046, which suggest that it is very likely that there is no essential difference between the two round of Delphi survey from a statistical perspective. What more, the concordance rate regarding to the score of importance of the statement is >80% in both the first and second round Delphi survey. Given its importance, we included the item in our final list. So a total of 43 statements were included in the framework.

### Results of survey of public health emergencies competences in public health students

The demographic information of 441 participants involved in the survey is shown in Table 3. A total of 168 (38%) study subjects were male and 273 (62%) were female. The number of students (82.84%) enrolled from the year of 2020, 2019, and 2018 are 219 (50%), 141 (32%), 77 (18%), respectively. 194 (44%) students are from urban area. As for distribution of speciality, the proportion of participants from preventive medicine, health inspection and quarantine, public health administration is 75, 15, 10%. The employment options of undergraduate students are also listed in Table 3. The top one job option is center for disease prevention and control (37%), followed by government offices (18%), hospital (6%), enterprise (6%). It is worth noting that a considerable proportion of students (29%) choose postgraduate education after undergraduate study.

**Table 3:** Demographic characteristics of participants (*n* = 441).

Demographic characteristics	*n* (%)	Intellectual competence	*p*	Practical competence	*p*	Behavioral competence	*p*	Personnel and motivation	*p*	Total competence	*p*
Total	441	6.33 (1.40)		9.60 (2.16)		50.29 (7.60)		25.93 (4.16)		92.15 (13.82)	
Sex			0.003		0.005		0.004		0.015		0.002
Male	168 (38)	6.58 (1.38)		9.95 (2.23)		51.55 (7.78)		26.49 (4.4)		94.58 (14.44)	
Female	273 (62)	6.18 (1.39)		9.38 (2.08)		49.52 (7.39)		25.58 (3.97)		90.66 (13.23)	
Residence			0.1		0.037		0.832		0.532		0.747
Urban area	194 (44)	6.48 (2.01)		9.89 (2.08)		50.64 (7.53)		25.85 (4.07)		92.86 (13.56)	
Rural area	247 (56)	6.21 (1.88)		9.73 (2.19)		50.02 (7.65)		26 (4.24)		91.6 (14.03)	
School year			<0.001		<0.001		<0.001		0.008		<0.001
~2018	81 (18)	6.74 (1.47)		10.31 (2.13)		53 (7.44)		27.27 (4.07)		97.32 (13.76)	
2019	141 (32)	6.57 (1.38)		10.11 (1.95)		50.35 (8.48)		25.88 (4.75)		92.71 (15.51)	
2020	219 (50)	6.04 (1.33)		9.01 (2.16)		49.27 (6.85)		25.47 (3.70)		89.80 (12.17)	
Professional background			0.105		0.391		0.713		0.088		0.718
Preventive medicine	330 (75)	6.32 (1.46)		9.63 (2.21)		50.45 (7.99)		26.15 (4.37)		92.55 (14.63)	
Health inspection and quarantine	66 (15)	6.61 (1.15)		9.76 (1.95)		49.59 (6.64)		25.15 (3.53)		91.55 (11.69)	
Public health administration	45 (10)	6.08 (1.23)		9.24 (1.88)		50.41 (5.58)		25.35 (3.18)		91.08 (9.92)	
Employment intention			0.165		0.011		0.224		0.480		0.153
Postgraduate education	129 (29)	6.57 (1.43)		10.12 (2.08)		51.26 (8.11)		26.4 (4.48)		94.35 (14.79)	
Hospital	26 (6)	6.31 (1.05)		9.77 (1.77)		51 (7.76)		25.81 (4.3)		92.88 (13.16)	
Disease control center	162 (37)	6.3 (1.37)		9.43 (2.22)		49.95 (7.06)		25.88 (3.88)		91.56 (12.9)	
Government office	80 (18)	6.15 (1.37)		9.31 (2.11)		49.91 (7.92)		25.66 (4.3)		91.04 (14.36)	
Enterprise	25 (6)	6.44 (1.33)		9.4 (1.76)		50.28 (6.37)		25.68 (3.61)		91.8 (11.44)	
Others	19 (4)	5.63 (1.8)		8.68 (2.79)		47.37 (8.29)		24.79 (4.3)		86.47 (14.96)	

The score was presented as *x* ± s̄ for the different domains of competences.

The performance of public health emergencies competences in public health undergraduate students was listed in Table 4. We used a 5-point scale ranging from 1 to 5 where 1 point indicated poor competency, while 5 point indicated excellent competency. The average score of the two indicators in intellectual competences was 3, with min and max score of 1 and 5 point, respectively. The two indicators are courses and disciplines, standards and regulations, which correspond to the domain of intellectual competences in the framework. Similar results were observed in practical competences with an average score of 3 for field investigation, information management and timely carry out the epidemic situation judged. The three indicators covers proposed abilities in such as case investigation, on-site hygiene survey and risk evaluation, sample collection and transportation/delivery, data analysis and management, report writing, identification of disease causality and chain of transmission in the framework.

**Table 4:** Performance of public health emergencies competences in public health undergraduate students.

Domain	Indicators	Score (5-point Likert scale)		
		Median	Min	Max
Intellectual competences	Subtotal	6	2	10
	Courses and disciplines	3	1	5
	Standards and regulations	3	1	5
Practical competences	Subtotal	9	3	15
	Field investigation	3	1	5
	Information management	3	1	5
	Timely carry out the epidemic situation judged	3	1	5
Behavioral competences	Subtotal	50	14	70
	Flexibility, agility and adaptability	3	1	5
	Systems and critic thinking	3	1	5
	Risk identification	3	1	5
	Physical fitness	3	1	5
	Resilience, effectively manage stress	4	1	5
	Communications	4	1	5
	Execution	4	1	5
	Situationally aware and contextual understanding	4	1	5
	Teamwork and Partnership	4	1	5
	Leadership	3	1	5
	Lecture and presentation	3	1	5
	Big picture thinking	4	1	5
	Dedication for working	4	1	5
	Sense of discipline and boundaries	4	1	5
Personnel and motivation	Subtotal	19	5	25
	Carefulness in work	4	1	5
	Innovation	3	1	5
	Actively learns	4	1	5
	Responsibilities	4	1	5
	Professional identity	4	1	5

As for behavioral competences, modest performance was observed in flexibility, systems and critic thinking, risk identification, physical fitness, leadership, lecture and presentation, with an average score of 3 for each field, which reflect proposed competence of professionalism and comprehensive personnel quality in the framework. While performance exhibited significant improvement in resilience, communications, execution, big picture thinking, dedication to working, sense of discipline and boundaries with an average score of 4 for each field, which are linked with proposed competence of emotion, attitude, and value in the framework. All the indicators, which reflect performance of personnel and motivation in the framework, are observed with an average score of 4 except for innovation. The above survey results indicated that students’ evaluation of their own motivation and attitudes is obviously better than the other domains such as knowledge and skills in the framework.

We further explored the possible relationship between competence performance and its determinants in this study. The performance distribution of the all the four competence domains was listed according to different factor groups as shown in Table 3. A significant difference in competence performance was observed between female and male participants. Male participants exhibited better performance than females in all the four competence domains, which were with an average total score of 94.58 and 90.66 (*p* = 0.002) respectively. The detailed frequency distribution of scores in both the female and male groups was presented in Supplementary Figure 1. Additionally, scores differed across school year groups, with a decrease trend from 2018 to 2020. The total average performance scores were 97.32, 92.71, 89.80 for the year of ~2018, 2019, 2020 (*p* < 0.001). The detailed frequency distribution of scores of different groups was presented in Supplementary Figure 2. No difference was observed in score distribution of residence, professional background, employment intention.

Multiple linear regression analysis found that sex and year of admission are independent influencing factors of competence performance as results shown in Table 5. The *β* of association between sex and overall performance is −0.141 (*p* = 0.003), while *β* of association between school year and overall performance is −0.237 (*p* < 0.001).

**Table 5:** Results of correlation analysis of emergencies competences and influencing factors.

Demographic characteristics	Intellectual competence		Practical competence		Behavioral competence		Personnel and motivation		Total competence	
	Standardized coefficients *β*	*p*	Standardized coefficients *β*	*p*	Standardized coefficients *β*	*p*	Standardized coefficients *β*	*P*	Standardized coefficients *β*	*p*
Sex	−0.145	0.002	−0.133	0.004	−0.134	0.005	−0.108	0.025	−0.141	0.003
Residence	−0.097	0.037	−0.128	0.005	−0.040	0.404	0.014	0.775	−0.47	0.316
School year	−0.277	<0.001	−0.307	<0.001	−0.215	<0.001	−0.144	0.018	−0.237	<0.001
Professional background	0.096	0.058	−0.084	0.091	0.062	0.230	−0.025	0.635	0.049	0.336
Employment intention	−0.015	0.789	−0.042	0.430	−0.001	0.981	−0.013	0.820	−0.013	0.819

## Discussion

We developed a framework of 43 core competences across four domains for public health emergencies response training in Fujian province using the Delphi method. The framework represent the current competence demands of public health work force to be qualified for their job roles in public health emergencies and may help to address the program training needs of local government in Fujian province.

This is the first attempt to develop a framework of core competences training requirements for health emergency medical students in educational programs. There have been previous efforts to discuss improvement of medical students’ ability in public health emergencies management and control in China. Fang et al. (18) published a systematic review which included 15 eligible studies quantitatively reported the current knowledge and skill level of medical students, and addressed the potential training needs for public health emergencies in China. However, data of the published studies was collected based on questionnaire surveys, and the questionnaires were self-designed by the investigator and with the suspicious of arbitrary (19). Many of the questions and indicators used were designed to depend on a students’ perspective rather than on expert opinion and requirements of professional work (20–22). The previous investigation put focus on the delivery of knowledge and training of basic skills, implement of field study practice, but neglect of behavioral competences and personnel and motivation (23, 24). In addition, most of the studies targeted general medical education and put special attention on clinical medicine instead of public health major (25, 26). And most of the studies included medical students in grades 1 to 4, which is not appropriate (27, 28), since students from lower academic years have not received any professional training of knowledge and skills in public health.

The core competency framework developed by the Delphi survey in this study covers part of the competences and domains in the ASPHER’s European List of Core Competences for the Public Health Professional (29). The first two domains of intellectual and practical competences are educational competences or professional competences, which delineate the knowledge and skill, as well as abilities that students are expected to achieve through diverse academic programs. They are organized around traditional academic disciplines like biostatistics, epidemiology, environmental health, social and behavioral sciences. The framework also covered indicators from behavioral competences and personnel and motivation, and these are considered to be fundamental determinants for accomplishment of individual and team task in the workplace.

The framework has effectively linked job requirements with the training of students. According to the core competency framework, a questionnaire was designed and competences listed in the framework was evaluated in newly graduated students from the school of public health in the survey. A total of 24 items was included in the survey which covered all the four scales of competency statement. According to the study results, the average performance of intellectual and practical competences is 3 in a 5-point scale, which equals 60 converted to 100 points. The results consist with the previous studies in other areas of China. Results from Fang et al. (18) study showed that the average score representing undergraduate students’ performance in knowledge related to public health emergencies ranged from 52.13 ± 8.17 to 79.43 ± 10.40 (10). These studies demonstrated that participants generally are inadequate in terms of knowledge and practical skills in public health emergencies. However, participants exhibited much better performance in indicators of behavioral competences and personnel and motivation with an average score of 4 for most of the items, which equals 80 converted to 100 points. Results from the survey indicate that the proposed competency framework can give a more comprehensive assessment of the potential of trainees in emergency response, compared with criteria used in other published studies. If the framework only includes intellectual and practical competences, the study would underestimate the potential of trainees with an average score of 3 in a 5-point scale. Because participants get significantly better performance in behavioral competences and motivation, which are very important quality in emergency response.

Finding from the current and published investigation suggest that medical colleges students have high-evaluation of their general competencies responding to public health emergency, but they do not have sufficient technical competencies such as practical knowledge and skills in field investigation, and their crisis awareness also needs further improvement. Thus, competency-based education is suggested for the core curriculum, and a functioning training resource platforms and program should be rebuild to provide core curriculum settings and teaching material. Educational strategy should be reinforced through formal training and on-the-job experience.

We also explored factors correlated with the knowledge, skills and attitude of medical university students in response to public health emergencies. The most significant determinant is the year of admission of participants. Substantially changes in medical education have happened due to the COVID-19 pandemic (30). The government implement transformative developments including competency-based education, interdisciplinarity, digital information technology and online teaching. Although these reforms were not originally initiated by the COVID-19 pandemic, the disease fortified their enforcement. Moreover, these changes are likely to exert a long-term effect on medical education. Given the increasingly severe public health challenges, governments at all levels in China have also increased their investment in public health emergency response training. Universities and experts from related fields have also discussed reform of the emergency response training system for students from public health, which include curriculum reconstruction, revision of the internship program, updating of teaching materials, and diversified teaching workforce. The school year was found to be an independent influencing factor of competences performance. A strong association was found between the school year and intellectual, practical, behavioral competences, as well as personnel and motivation. However, the performance was not improved after the breakout of COVID-19 as expected. Compared with participants enrolled in the year of 2018 or before, scores of participants from 2019 and 2020 significantly decreased across all the four competence domains with the greatest attenuation observed in practical competence, followed by intellectual, behavioral competence and motivation. One of the possible explanations is that the epidemic has seriously disrupted the routine of teaching and education of students. A large number of courses have been shifted to online teaching, which has greatly compromised the teaching quality and effectiveness, leading to a significant compromise in the practical learning and theoretical study (31). On the other hand, online teaching at home also significantly reduced the communication and learning initiatives of students, thus considerably harming the development of behavioral competences, personality and motivation. The effects of transformative developments in health emergency response training before and after COVID-19 pandemic may still need years ahead to evaluate and manifest.

Another important determinant of performance is gender. Actually for the department of public health, the proportion of female student has largely increased in the last two decades. The proportion of female student exceeds that of male student, as can be reflected by data from the current study with 63% of participant are female. And this proportion is even more pronounced in postgraduate education. Though the number of female students overwhelmed male, the competences performance of male student is significantly better than female students in all competences domains including intellectual, practical, behavioral competences as well as personnel and motivation. According to a reported meta-analysis of gender equity in the health workforce from 104 countries, 70% of workers in the health and social sector are female (32). Similar results were observed in another study of ours which explored influencing factors of scientific creativity and innovation ability, the average scores of creative thinking and academic performance is lower in female students (33).

There are several limitations of the study. It is a single center based survey and mainly relied on self-reported data, which would introduce bias to the result, and requires more work to recruit participants from other institutes and regions in the future studies to generalize the findings to other medical students from China. Additionally, the observed differences of determinants such as gender in competency performance are notable, but the study does not fully explore the underlying causes of these differences due to the fact that details of those factors was not collected in the study.

## Conclusion

A framework of 43 core competences was developed by Delphi method. The framework covered both technical and general competencies in public health emergencies and represent the current competence demands of public health work force to be qualified for their job roles in public health emergencies for the local government in Fujian province. The concordance rate regarding to score of importance of the core competences are all >80% in both the first and second round Delphi survey, suggesting considerable reliability of the framework. Additionally, Survey conducted and guided by the framework suggesting that undergraduate students generally have a low ability of knowledge and practical skills, while have a modest performance in behavioral competences and personnel and motivation in public health emergency.

## Data Availability

The raw data supporting the conclusions of this article will be made available by the authors, without undue reservation.
