# Supplementary figures and images for: Development of a core competence model for improving medical college students’ ability in respond to public health emergencies
Source: Front Public Health. 2025 Mar 20;13:1467832. doi: 10.3389/fpubh.2025.1467832 (PMC11965672; doi:10.3389/fpubh.2025.1467832)

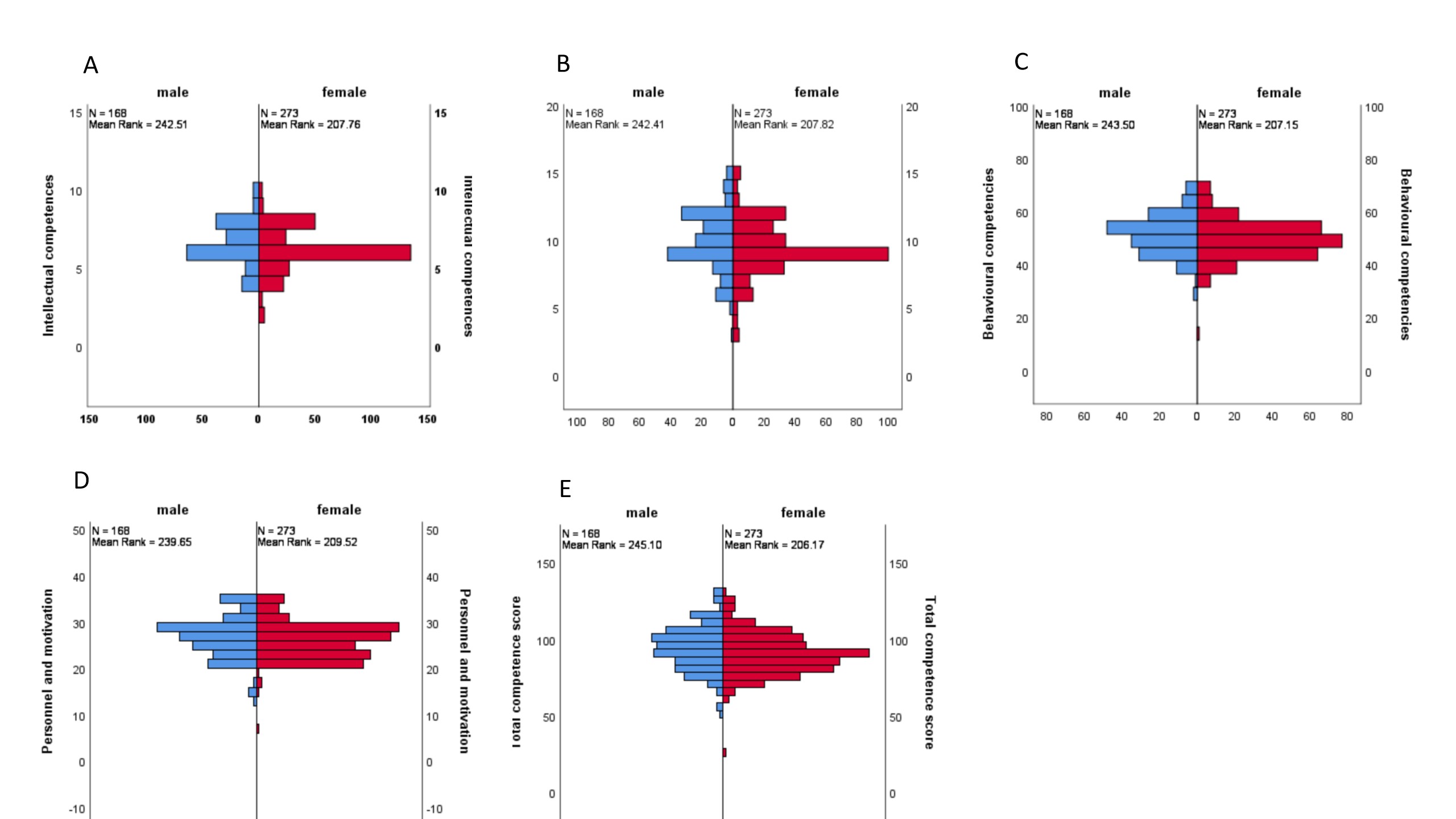

Supplement: Supplementary file 1 [file Image_1.jpeg]

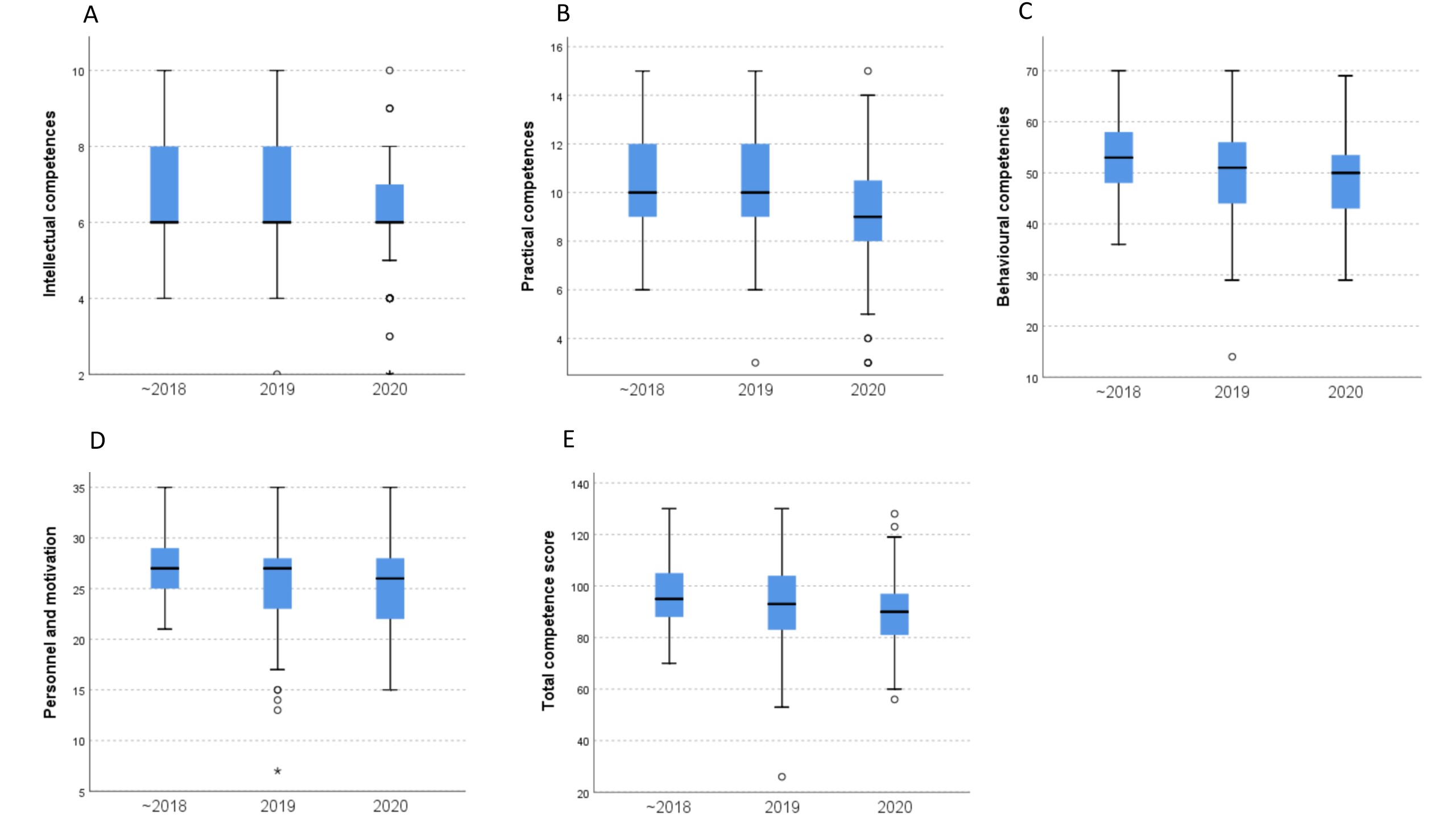

Supplement: Supplementary file 2 [file Image_2.jpeg]
